# Supplementary material for: Shifts in Fusarium Communities and Mycotoxins in Maize Residues, Soils, and Wheat Grains throughout the Wheat Cycle: Implications for Fusarium Head Blight Epidemiology
Source: Microorganisms. 2024 Aug 28;12(9):1783. doi: 10.3390/microorganisms12091783 (PMC11434071; doi:10.3390/microorganisms12091783)
Supplement: Supplementary file 1 [file microorganisms-12-01783-s001.zip › Figure S1. Calibration curves.pdf]

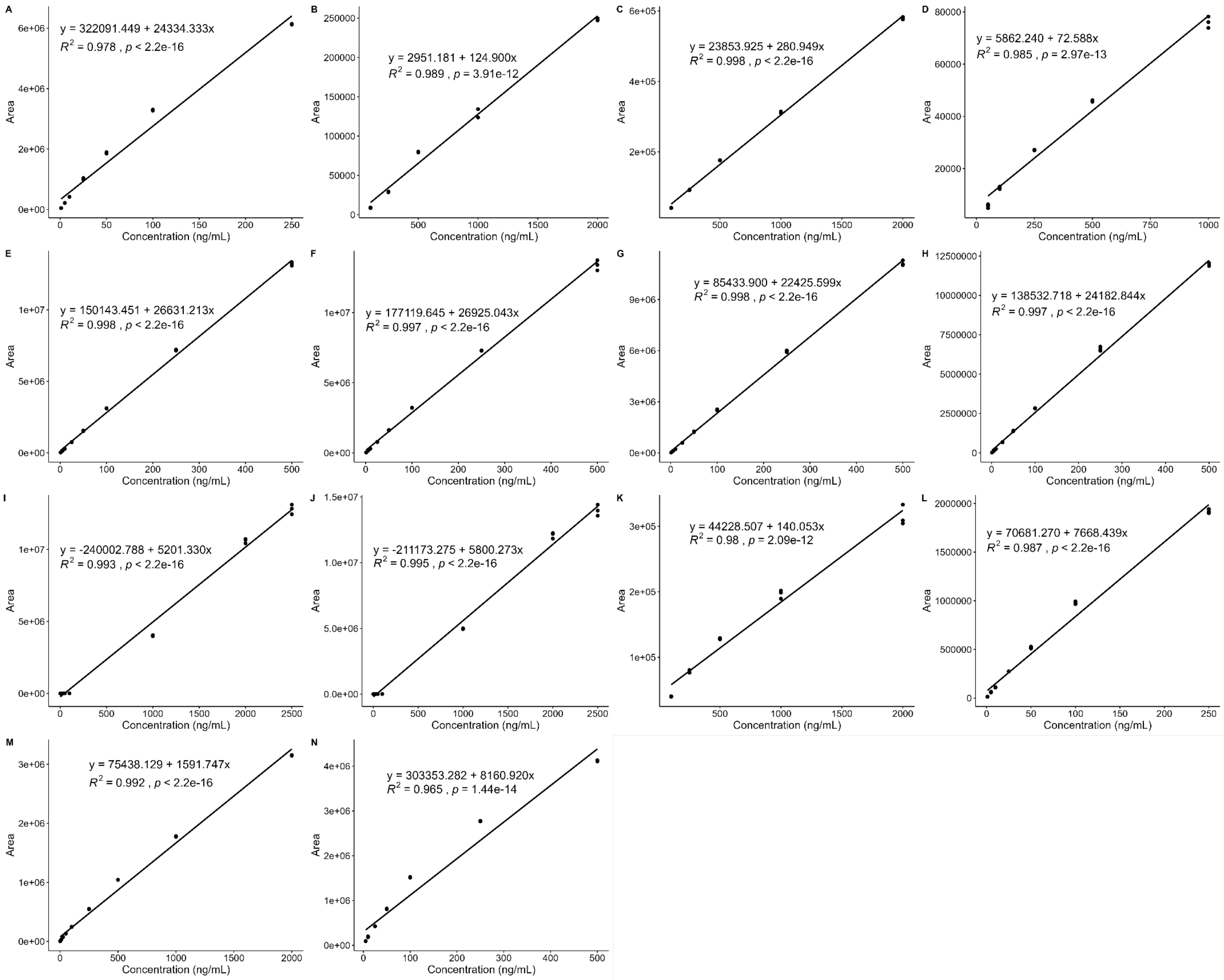
A
y = 322091.449 + 24334.333x
R^2 = 0.978 , p < 2.2e-16
B
y = 2951.181 + 124.900x
R^2 = 0.989 , p = 3.91e-12
C
y = 23853.925 + 280.949x
R^2 = 0.998 , p < 2.2e-16
D
y = 5862.240 + 72.588x
R^2 = 0.985 , p = 2.97e-13
E
y = 150143.451 + 26631.213x
R^2 = 0.998 , p < 2.2e-16
F
y = 177119.645 + 26925.043x
R^2 = 0.997 , p < 2.2e-16
G
y = 85433.900 + 22425.599x
R^2 = 0.998 , p < 2.2e-16
H
y = 138532.718 + 24182.844x
R^2 = 0.997 , p < 2.2e-16
I
y = -240002.788 + 5201.330x
R^2 = 0.993 , p < 2.2e-16
J
y = -211173.275 + 5800.273x
R^2 = 0.995 , p < 2.2e-16
K
y = 44228.507 + 140.053x
R^2 = 0.98 , p = 2.09e-12
L
y = 70681.270 + 7668.439x
R^2 = 0.987 , p < 2.2e-16
M
y = 75438.129 + 1591.747x
R^2 = 0.992 , p < 2.2e-16
N
y = 303353.282 + 8160.920x
R^2 = 0.965 , p = 1.44e-14
Area
Concentration (ng/mL)

Figure S1. Matrix-matched calibration curves used for the quantitation of tested mycotoxins. (A) Zearalenone, (B) Deoxynivalenol, (C) 15-acetyl-deoxynivalenol or 3-acetyl-deoxynivalenol, (D) Nivalenol, (E) Enniatin B, (F) Enniatin B1, (G) Enniatin A, (H) Enniatin A1, (I) Fumonisin B1, (J) Fumonisin B2, (K) Moniliformin, (L) Beauvericin, (M) HT-2 toxin, (N) T-2 toxin.
