# Supplementary material for: Shifts in Fusarium Communities and Mycotoxins in Maize Residues, Soils, and Wheat Grains throughout the Wheat Cycle: Implications for Fusarium Head Blight Epidemiology
Source: Microorganisms. 2024 Aug 28;12(9):1783. doi: 10.3390/microorganisms12091783 (PMC11434071; doi:10.3390/microorganisms12091783)
Supplement: Supplementary file 1 [file microorganisms-12-01783-s001.zip › Figure S2. EIC.pdf]

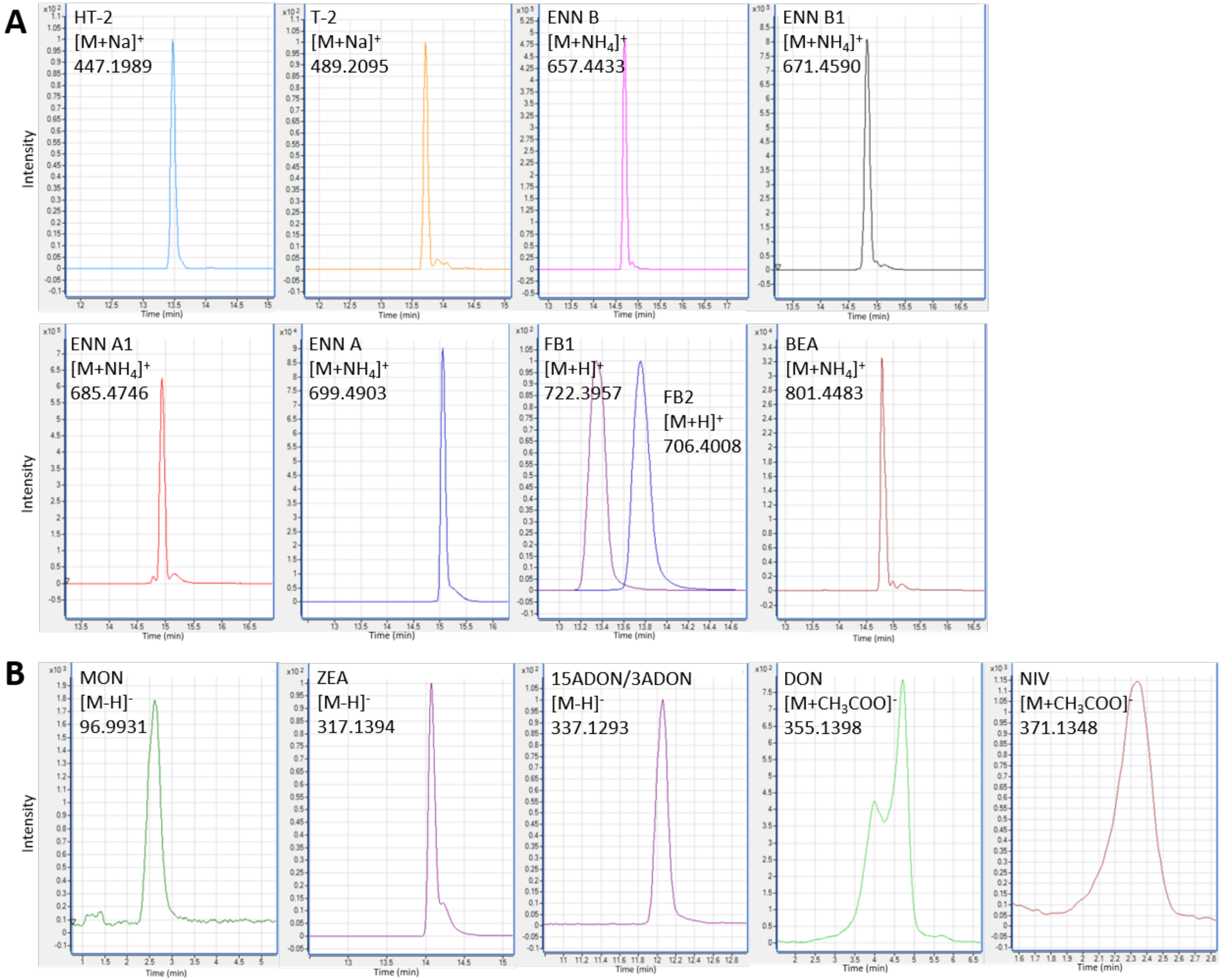

Figure S2. Extracted ion chromatograms (EIC) in positive (A) and negative (B) ion modes for a matrix-matched calibration standard of fifteen mycotoxins at 5000 ng/mL each. HT-2: HT-2 toxin; T-2: T-2 toxin; ENN B: enniatin B; ENN B1: enniatin B1; ENN A: enniatin A; ENN A1: enniatin A1; FB1: fumonisin B1; FB2: fumonisin B2; BEA: beauvericin; MON: moniliformin; ZEA: zearalenone; 15ADON: 15-acetyl-deoxynivalenol; 3ADON: 3-acetyl-deoxynivalenol; DON: Deoxynivalenol; NIV: nivalenol.
